# Bacteriophages from treatment-naïve type 2 diabetes individuals drive an inflammatory response in human co-cultures of dendritic cells and T cells

**DOI:** 10.1080/19490976.2024.2380747

**Published:** 2024-07-27

**Authors:** Torsten P. M. Scheithauer, Koen Wortelboer, Maaike Winkelmeijer, Xanthe Verdoes, Ömrüm Aydin, Yair I. Z. Acherman, Maurits L. de Brauw, Max Nieuwdorp, Elena Rampanelli, Patrick A. de Jonge, Hilde Herrema

**Affiliations:** a Experimental Vascular Medicine, Amsterdam UMC location AMC, Amsterdam, The Netherlands; b Diabetes & Metabolism, Amsterdam Cardiovascular Sciences, Amsterdam, The Netherlands; c Amsterdam Gastroenterology Endocrinology Metabolism, Amsterdam, The Netherlands; d Department of Surgery, Spaarne Hospital, Hoofddorp, The Netherlands; e Amsterdam UMC location AMC, Vascular Medicine, Amsterdam, The Netherlands; f Amsterdam Institute for Immunology and Infectious Diseases, Amsterdam, The Netherlands

**Keywords:** Bacteriophages, obesity, type 2 diabetes, inflammatory response

## Abstract

Individuals with type 2 diabetes (T2D) show signs of low-grade inflammation, which is related to the development of insulin resistance and beta-cell dysfunction. However, the underlying triggers remain unknown. The gut microbiota is a plausible source as it comprises pro-inflammatory bacteria, bacterial metabolites and viruses, including (bacterio)phages. These prokaryotic viruses have been shown to mediate inflammatory responses in gastrointestinal disease. Given the differences in phage populations in healthy individuals versus those with cardiometabolic diseases such as T2D, we here questioned whether phages from T2D individuals would have increased immunogenic potential. To address this, we isolated intestinal phages from a fresh stool sample of healthy controls and individuals with newly diagnosed, treatment-naive T2D. Phages were purified using cesium chloride ultracentrifugation and incubated with healthy donor dendritic cells (DCs) and autologous T cells. Donors with T2D had slightly higher free viral particle numbers compared to healthy controls (*p* = .1972), which has been previously associated with disease states. Further, phages from T2D induced a higher inflammatory response in DCs and T cells than phages from HC. For example, the expression of the co-stimulatory molecule CD86 on DCs (*p* < .001) and interferon-y secretion from T cells (*p* < .01) were increased when comparing the two groups. These results suggest that phages might play a role in low-grade inflammation in T2D individuals.

## Introduction

Obesity and type 2 diabetes (T2D) are growing challenges for health care systems worldwide.^1^ Both are associated with low-grade inflammation that is disturbing the function of several metabolic tissues, including adipose tissue, skeletal muscle, liver, and pancreas.^2^ Inflammation reduces insulin sensitivity^3^ and ultimately leads to the development of beta-cell dysfunction,^4^ thereby leading to the reduced insulin activity seen in T2D.^5^ Tissue inflammation often coincides with increased activity and accumulation of pro-inflammatory immune cells.^6^ The primary triggers for this inflammatory response are plentiful, with the gut microbiota being a plausible source of such triggers.^7^

The intestine harbors various types of microorganisms and viruses.^8^ Alterations in bacterial composition and functional potential have been related to various diseases.^9^ An increase in pro-inflammatory *Proteobacteria* have for example been associated with obesity^10^ and T2D.^11^ This group of bacteria is known to produce lipopolysaccharide (LPS) that can pass the intestinal barrier and induce inflammation.^12^ LPS has been related to the low-grade inflammation seen in T2D.^13^ Several other bacterial metabolites are related to inflammation in metabolic diseases.^7^ Yet, the role of viruses, particularly those targeting bacteria (bacteriophages), in metabolic inflammation is mostly unexplored.

Bacteriophages, also called phages, are highly abundant viruses that infect bacteria.^14^ Changes in the intestinal virome have been noted in various diseases.^15^ For example, the gut virome differs between healthy individuals and those with inflammatory bowel disease (IBD).^16^ Interestingly, isolated phages from individuals with IBD were able to induce an inflammatory response in the eukaryotic host.^17^ Phages were shown to activate dendritic cells (DCs) via toll-like receptor (TLR) 9 resulting in the propagation of interferon-y (IFN-y)± T cells which exacerbated colitis development in mice.^17^

Alterations in the gut virome have also been noted in obesity^18,19^ and T2D.^20,21^ Interestingly, transplantation of the phageome of lean mice into obese mice altered gut microbiome composition and improved the recipients’ glucose tolerance.^22^ We recently showed that fecal filtrate transplantation from lean healthy donors to recipients with metabolic syndrome was safe, altered gut phage-bacteria dynamics and improved time in the range for glucose.^19^ Whether phages from healthy individuals had reduced inflammatory potentially compared to residing phages in recipients with metabolic syndrome was beyond the scope and limitations of the human intervention study. We therefore set out here to conceptually address the pro-inflammatory potential of gut viruses from healthy and T2D donors *in vitro*. We included 12 healthy individuals and 12 individuals with newly diagnosed T2D (treatment-naïve). We used *de novo* metagenomics approaches to characterize the gut virome (both virions and prophages) in both groups. Isolated virions from fresh stool of healthy and T2D donors were used to stimulate primary healthy donor immune cells using a co-culture approach. We show that virions from T2D donors have enhanced immunogenic potentially compared to virions form lean healthy donors.

## Methods

### Human participants

Healthy individuals were included within the PIMMS study in Amsterdam (NL67136.018.18). Individuals with newly diagnosed T2D were included within the BARIA cohort Amsterdam (NL55755.018.15). Fresh feces were collected from the participants, and fecal viruses were directly isolated on the same day within 6 hours of defecation. In addition, aliquots of the feces were collected and directly stored at −80°C for later bulk metagenomic sequencing. The study protocols were approved by the Ethical Review Board of the Academic Medical Center, Amsterdam and all patients that have been included provided informed consent. Characteristics of the participants can be found in Table 1.

**Table 1.** Clinical characteristics of participants.

	ND	*de novo* T2D	*p*-value
Sex (M/F)	6/6	5/7	.6977
Age (years)	36.9 ± 12.5	54.4 ± 17.9	.0011
HbA1c (mmol/mol)	34.8 ± 10.3	51.2 ± 20.2	<.0001
T2D medication	No	No	N/A
BMI (kg/m^2^)	23.4 ± 2.8	41.6 ± 14.5	<.0001

Abbreviations: M male, F female, HbA1c glycated hemoglobin, T2D Type 2 diabetes, BMI body mass index.

### Free fecal phage isolation

Fresh fecal samples (>50 g) were diluted and homogenized in sterile saline (three parts fecal weight). Samples were centrifuged for 10.000 × g for 60 min (4°C) for two times to separate debris and bacteria from phages. The supernatant was filtered through a sterile 0.2 µm PES membrane using a tangential flow filtration system (Vivaflow® 50, Sartorius AG, DE). Fecal filtrates with phages were stored at 4°C until further use.

### Toll-like receptor ligand measurements

TLR ligands in fecal filtrates were measured with Human Embryonic Kidney (HEK)-Blue 293 cell line TLR reporter cell lines, which express a specific human TLR gene and an NF-κB-inducible SEAP (secreted embryonic alkaline phosphatase) reporter gene (Invivogen, US). Samples were diluted in ultrapure water (ThermoFisher, US) and incubated with TLR2, TLR4, TLR5, and TLR9 reporter cells for 16 h according to manufacturer’s instructions.

### LPS measurement

Lipopolysaccharide (LPS) was measured with the Pierce^TM^ Chromogenic Endotoxin Quant Kit, a Limulus Amebocyte Lysate (LAL) assay, according to the manufacturer’s instructions (Thermo Fisher, US).

### Purification of fecal filtrates

Fecal phages were purified from bacterial metabolites and debris, including LPS, via ultracentrifugation according to Lawrence et al (2010).^23^ In brief, fecal filtrates were applied to a cesium chloride gradient. Samples were spun for 3 h at 60.000 × g (4°C). The 1.5 g/mL layer was taken with a syringe and applied to a 10 kDa Amicon filter unit. Phages were washed three times with SM buffer (2500 × g, 5 min, 4°C). Purified phages were stored at 4°C until further use.

### Phage counts

VLPs were measured with fluorescence microscopy according to Thurber et al. (2009).^24^ In brief, fecal filtrates or purified phages were added to a 0.02 µm filter. The filter with the phages was stained with SybrGold (ThermoFisher, US). Pictures were taken with a Leica DM6 fluorescent microscope. VLPs were counted with ImageJ.

### Human monocyte/CD4 T cells isolation and DC differentiation

Peripheral blood was collected in EDTA vacutainers (BD, US). Peripheral blood mononuclear cells (PBMCs) were isolated with Lymphoprep^TM^ (Stemcell technologies, US) according to manufacturer’s instructions. CD14-positive cells (monocytes) were isolated with the MACS® cell separation technology (Miltenyi Biotics, DE), using CD14 microbeads and a manual magnet. For differentiation, 3 × 10^6^ cells per well were seeded in a 6 well suspension plate in complete RPMI. Culture media is composed as following: RPMI 1640 (ThermoFisher, US), 10% FBS (Capricorn Scientific, DE), 1× GlutaMax (ThermoFisher, US), 1× pen/strep (ThermoFisher, US). Cells were incubated for 2 days with 800 IU/mL of human granulocyte-macrophage colony-stimulating factor (GM-CSF) and 250 IU of human IL-4 (Miltenyi Biotec, DE). Half of the media was changed and fresh cytokines were added for another 4 days.

CD4 positive cells (T cells) were isolated from the same donor PBMCs with the aid of CD4 microbeads following the manufacturer’s instructions (Miltenyi Biotec, DE). The culture media with the phages was saved for cytokine analysis and 500.000 T cells per well were added.

### Phage stimulation of DC in mono/co-culture with T cells

Monocytes derived from DCs were seeded at 100,000 cells per well in a 24 well plate. Cells adhered to the plate for 24 h. The same protocol as described in Gogokhia et al. (2019) was used for co-culture.^17^ Purified fecal phages were added to the cells for 24 h and the supernatant was stored at-80C for ELISA assay. For DC:T cells co-culture, 500.000 T cells per well (containing 100,000 DCs) were added after washing with PBS. The co-culture was incubated for another 72 h. Supernatant was saved and RNA was isolated from cells.

### ELISA

IL-6 and IFNg secretion were measured in cell supernatants using the human IL-6/IFNgamma DuoSet ELISA kit (R&D Systems), according to the manufacturer’s procedures.

### RNA isolation and cDNA synthesis

RNA was isolated with TriPure^TM^ isolation reagent (Roche, CH). The cells were separated from the culture media and 300 uL TriPure^TM^ was added. After lysis, 60 uL chloroform was added, the mixture was vigorously shaken for 15 seconds and incubated for 3 minutes at room temperature. Next, the samples were spun for 15 minutes at 12.000 × g (4°C) and the aqueous phase was mixed with 190 uL isopropanol with 0.44 uL GlycoBlue^TM^. After an overnight incubation at −20°C, samples were spun at 12.000 × g for 10 minutes (4°C) and the pellet was washed twice with 1 mL of 75% ethanol (7.500 × g, 5 minutes, 4°C). Next, the pellet was dried at room temperature for 10 minutes, 18 uL RNase free H2O was added and incubated at 56°C for 10 minutes. RNA concentration was measured with Nanodrop. cDNA synthesis was performed with SensiFAST^TM^ cDNA Synthesis Kit (Meridian Bioscience, US) according to manufactures instructions.

### Gene expression analysis

Gene expression was measured *via* real-time quantitative PCR (RT-qPCR) with the aid of PCR machine (BioRad, US). SensiFAST^TM^ SYBR® No-ROX Kit was used according to the manufacturer’s instructions. For each well, 7.5 ng cDNA and 1 μM primer mix (Table 2) were used in a 10 uL PCR mix. The following PCR program was used: 95°C for 10 minutes, 40 cycles of 95°C for 15 seconds and 60°C for 30 seconds with a plate reading, followed by a melt curve with an increment of 0.5°C every 5 seconds starting from 65°C to 95°C.

**Table 2.** Primer sequences.

Name	Species	Forward	Reverse	Ref.
18S	human	GAGGGAGCCTGAGAAACGG	GTCGGGAGTGGGTAATTTGC	This study
IL6	human	CTG CAG AAA AAG GCA AAG AAT CTA	GTT GTC ATG TCC TGC AGC C	This study
IFNγ	human	ACCAGAGCATCCAAAAGAGTGT	GGACATTCAAGTCAGTTACCGAATA	This study

### FACS analysis and gating of DCs

Monocyte derived DCs were incubated with control, and purified phages of T2D and ND donors as mentioned above. Cells were collected and stained for phenotyping with Fluorescent-activated cell sorting (FACS) using antibodies anti-HLA-DR, CD86, and CD80 (BD Biosciences, Table 3). Surface expression at single-cell levels was measured on a FACS-Canto machine (Becton & Dickinson), and data were analyzed using FlowJo software (TreeStar) according to the gating strategy (Figure S5).

**Table 3.** Antibodies used for FACS.

Name	Fluorescence	Company	Ref. number	Lot. number
HLA-DR	PerCP-Cy5.5	BD Biosciences	560652	9074606
CD86	FITC	BD Biosciences	555657	7348597
CD80	PE	BD Biosciences	557227	8005921

### DNA isolation

Total genomic DNA was extracted from the frozen fecal aliquots using a repeated bead-beating method as previously described.^25^ Briefly, 250 mg of feces was homogenized with 700 µl of S.T.A.R. buffer (Roche, CH) in bead-beat tubes using a bead-beater (FastPrep-24^TM^, MP Biomedicals, US) set to three times 5.5 ms for 1 minute. Homogenates were further lysed by incubation at 95°C for 15 minutes, whereafter the samples were centrifuged for 5 minutes at 14,000 × g, 4°C, and supernatant transferred to nuclease-free tubes. To extract any remaining DNA from the bead-beating tubes, 300 µl of S.T.A.R. buffer was added to the pellet and the above steps were repeated. Subsequently, the DNA was cleaned using the Maxwell® RSC Blood DNA Kit (Promega, US) according to the manufacturer’s instructions, and stored at −20°C until library preparation.

In addition, DNA from the purified phage virions was isolated as previously described.^19^ Briefly, purified virions in the SM buffer were treated with DNases to digest any free-DNA debris, whereafter the virions were lysed by incubation with sodium dodecyl sulfate (SDS) and proteinase K at 56°C for 1 hour. DNA was extracted from the lysates using a two-step phenol/chloroform extraction protocol, followed by an additional round of purification using the DNeasy Blood&Tissue kit (Qiagen, NL) according to the manufacturer’s protocol.

### Metagenomic sequencing

Libraries for both bulk and VLP metagenomic sequencing were prepared using the NEBNext Ultra II FS DNA library prep kit with NEBNext Multiplex Oligos for Illumina (New England Biolabs, US) according to the manufacturer’s instructions. For bulk DNA, fragmentation with the FS enzyme mix was performed for 10 minutes, while fragmentation time was 5 minutes for VLP DNA samples. In addition, due to the low input DNA concentrations for the VLP samples, the NEB adaptors for Illumina were diluted 10 times to prevent dimer formation. Following adapter ligation, DNA fragments of 300–500 bp were purified and amplified with 10 PCR cycles for low concentration VLP samples, while bulk DNA was amplified with the minimal 3 PCR cycles. After clean-up of the PCR reaction, quality, and concentration of the bulk and VLP libraries where assessed by Qubit with the dsDNA HS kit (Thermo Fisher Scientific, US) and Agilent High Sensitivity D5000 ScreenTape system (Agilent Technologies, US). Sequencing was performed on an Illumina NovaSeq 6000 platform using 2 × 150 bp paired-end chemistry with the S4 Reagent Kit v1.5 (300 cycles) at the Core Facility Genomics of Amsterdam UMC.

### Bioinformatic analysis

#### Assembly

Trimming and quality control of metagenomic sequencing reads of both the WGS and VLP datasets was done with fastp v0.23.3.^26^ Tadpole (options mode=correct, ecc=t, prefilter = 2) was used for error correction, and clumpify (options dedupe=t, optical=t, dupedist = 12000) for deduplication. Both programs are from bbmap v38.90 [https://jgi.doe.gov/data-and-tools/bbtools]. Reads were then assembled with metaSPADES v3.15.5.^27^

### Viral sequence recognition

Viral sequences among the assemblies were identified using virsorter v2.2.3 (option – exclude-lt2gene)^28^ and checkv v1.0.1.^29^ Contigs fulfilling at least one the following criteria were taken to be viral: >1 checkv-identified viral gene, ≥0.95 viral score from virsorter, ≥2 virsorter-identified viral hallmark genes, no viral or bacterial genes identified by checkv. In total, this identified 11,371 viral contigs, of which 10,893 were left after removing duplicate sequences with bbdupe from bbmap v38.90 (option minidentity = 100). Non-redundant viral sequences were subsequently clustered into viral populations (VPs) at 95% average nucleotide identity (ani) with the checkv-related aniclust.py script.^29^ Viral host were predicted with iPHoP v1.3.2.^30^

### vOTU table construction

HQ-reads were subsequently mapped to the non-redundant phage sequences with bowtie2 v2.4.2.^31^ Reads that mapped with less than 90% ani were removed with coverm filter v0.6.1 (option – min-read-percent-identity 90, https://github.com/wwood/CoverM). Horizontal read coverage was determined using samtools idxstats v1.15.1,^32^ and read depths of contigs with a horizontal coverage of less than 75% were set to zero. Read counts were then summed per VP, and differential contig lengths were taken into account by calculating reads per kilobase per million mapped reads (RPKM) values.

### Bacterial analyses

Bacterial community profiles were determined with mOTUs v3.0.3.^33^ Metagenome-assembled genomes (MAGs) were constructed from the WGS dataset as follows: HQ reads were mapped to assembled scaffolds that were longer than 2500 bp with bowtie2 v2.4.2. After constructing read depth tables with jgi_summarize_bam_contig_depths v2.15, contigs were binned using metabat2 v2:2.15.^34^ The quality of MAGs was assessed with checkm v1.2.1,^35^ and MAGs were kept for subsequent analysis if completeness - (5 × contamination) was at least 50. The MAG taxonomy was determined with GTDB-Tk v2.1.1^36^ using the R207-v2 database package.

### Statistical analyses

All statistical analyses were performed in R v4.3.0. Ecological measures and ordinations were calculated with the vegan v2.6–4 R package.^37^ The former used RPKM values, while the latter used centered log-ratio (CLR) transformed data. The VPs with RPKM < 100 across all samples or >20 in at least 10% of the samples were discarded before analysis. The PERMANOVA tests were calculated using vegan and adjusted for age and sex. Differential abundance analyses were done with ANCOM-BC v1.2.2^38^ on the raw read counts, as this method implements its own normalization and adjustment for multiple testing. ANCOM-BC was run with structural zero discovery was turned on, but the usage of the asymptotic lower bound turned off and corrected for age and sex.

### Data availability

The data that support the findings of this study are available in ENA accession# PRJEB74045 (access can be granted upon request with corresponding author HH).

## Results

The gut microbiota of individuals with T2D differs from healthy individuals.^11^ In addition to metabolic status, oral medication like the diabetes drug metformin^39^ strongly influence the microbiome.^40^ We therefore opted to include only individuals with newly diagnosed T2D (treatment-naïve, *n* = 12) and healthy controls (*n* = 12). Clinical characteristics of the donors are depicted in Table 1. The majority of T2D individuals in our diabetes clinic is obese and middle-aged making it challenging to recruit lean, non-medicated healthy controls of similar age and BMI. Hence, we have significant age and BMI differences between the groups, for which we corrected in our analyses.

### Phages from diabetic individuals stimulate inflammatory crosstalk between dendritic cells and T-cells

**Figure 1.**
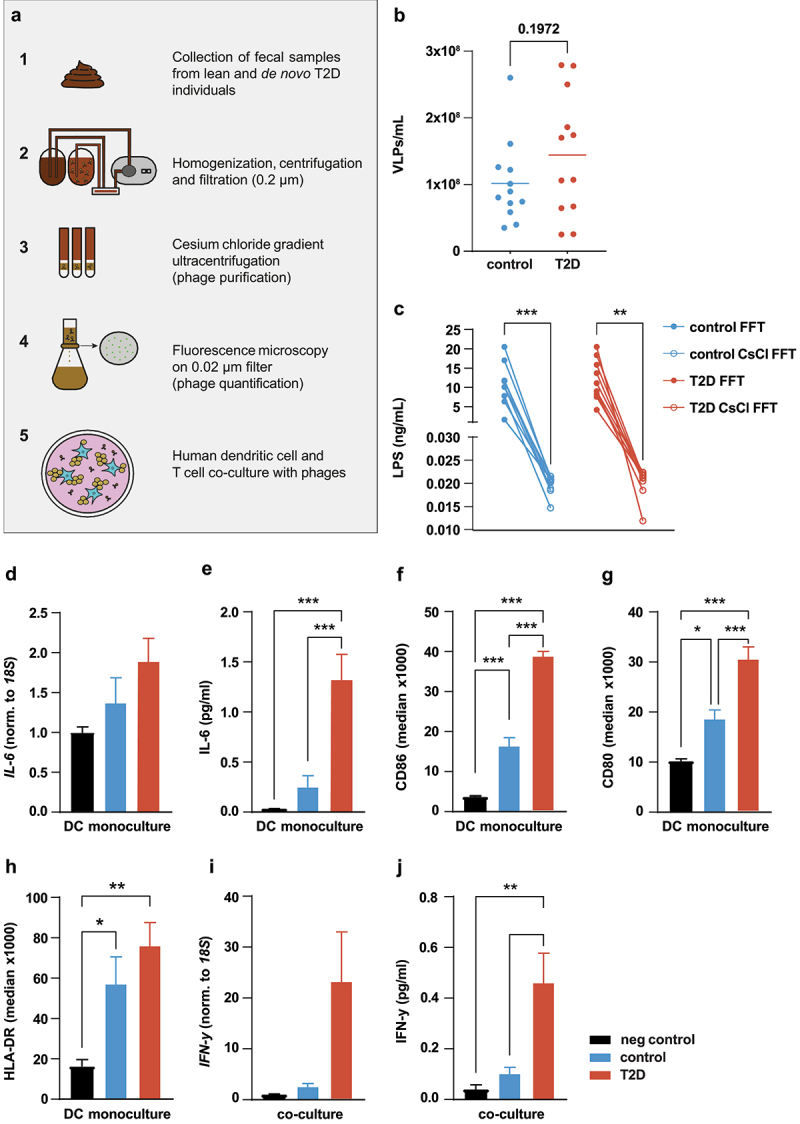
Increased inflammatory response by phages derived from individuals with T2D.

Fecal samples were freshly processed within 6 hours of defecation. Microorganisms were removed by centrifugation and subsequent tangential flow filtration (0.2 µm). Phages were further isolated and concentrated using cesium chloride (CsCl). Phages were stored at 4°C until further use (Figure 1(a)). Virus-like particles (VLPs) were quantified using fluorescence microscopy (for representative images of VLPs please see Figure S1 a, b). There was a non-significant trend towards more VLPs in T2D individuals than in healthy controls (Figure 1(b)), which was previously associated with worsened disease status in IBD.^17^

(a) Workflow. Fresh fecal samples were collected from individuals with type 2 diabetes (T2D) and healthy controls (*n* = 12 per group). Fecal samples were homogenized and phages were separated by (ultra)centrifugation as well as tangential filtration (0.2 µm) from bacteria and their metabolites. Free fecal phages were quantified with florescence microscopy. Healthy donor human immune cells were incubated with purified phages.

(b) Viral titer determined by fluorescent microscopy in VLPs per ml fecal filtrate (normalized for fecal input).

(c) LPS concentration before and after CsCl purification.

(d-j) Purified fecal phages were incubated with human dendritic cells for 24 h, followed by 72 h with human T cells. Cytokines were measured in cell culture supernatant and RNA expression was measured in the cells.

(d-e) IL-6 gene expression in and protein secretion from healthy donor dendritic cells.

(f-h) Cell surface markers CD86, CD80, and HLA-DR on healthy donor dendritic cells.

(i-j) IFN-y gene expression in and protein secretion from T cells.

Abbreviations: VLP, virus-like particle; LPS, lipopolysaccharide; T2D, type 2 diabetes; CsCl, cesium chloride; FFT, fecal filtrate. *p < .05, **p < .01, ***p < .001.

Gut-derived LPS is a strong immunogenic stimulus.^7^ Prior to using phage solutions in cell cultures, we measured LPS in the fecal filtrates and in CsCl-purified phage solutions. LPS was reduced more than 100-fold upon CsCl purification to an average of 0.02 ng/ml (Figure 1(c)). We also measured ligand abundance in purified phage samples for TLR3, TLR4, TLR5, and TLR9 with the aid of HEK-Blue TLR reporter cell lines. Receptor activation by phage samples was in the range of negative controls (Figure S1c-f), suggesting that the majority of free-floating immunogenic bacterial components were removed from the phage solutions.

Dendritic cells (DCs) are an important first-line defense in the mammalian intestine and as such activate the adaptive immune system, including T cells. Gogokhia et al. suggested an interplay between DCs and T cells as a plausible pathway in the response of the mammalian immune system to intestinal phages.^17^ We differentiated freshly isolated healthy donor monocytes to DCs and, to determine tolerable VLP-load, incubated them with increasing VLP numbers of a pooled phage solution for 24 h. Phages dose-dependently increased *IL-6* expression (Figure S2a) and IL-6 secretion (Figure S2b). As it was the lowest concentration to offer an effect, we used 10^7^ VLPs/mL, for all subsequent experiments. To check for the effect of any remaining LPS in the purified phage solutions, we stimulated DCs with *Escherichia coli*-derived LPS at a concentration of 0.1 ng/ml (10× higher than the LPS concentrations detected in the phage solution, Figure 1(c)). Interestingly, we observed significantly higher IL-6 protein secretion when DCs were stimulated with phage solution at a concentration of 10^7^ VLPs/mL compared to the LPS control (Figure S2c), underscoring that the pro-inflammatory response in DCs is induced by phages.

We then compared the effect of healthy- and T2D-derived phage solutions on monocytes-derived DCs. Phage solutions from 12 healthy and 12 T2D donors were pooled and incubated with human DCs for 24 h. Compared to healthy donor-derived phage solutions, those from individuals with T2D showed a trend toward increased *IL-6* gene expression (Figure 1(d)) and markedly higher IL-6 protein secretion from human dendritic cells (Figure 1(e)). The DC activation markers CD86 and CD80, as determined by FACS analyses (Figure 1(f,g)), were significantly more increased by phage solutions from T2D donors than controls, although the latter also resulted in significant increases. Meanwhile, HLA-DR (Figure 1(h)) was increased by both T2D and controls in the same manner. These results highlight that fecal phages are able to activate and induce an inflammatory response in dendritic cells.

Given the phage-induced upregulation of MHC-II and co-stimulatory molecules, we next addressed the putative effect of phages on DCs-CD4 T cell encounters. We pulsed healthy donor dendritic cells with pooled phage solutions from healthy and T2D donors for 24 hours. The phage-containing media was then removed and same-donor CD4 T cells were cocultured with the activated DCs for 72 h. Phage solutions from individuals with T2D induced significantly more *IFN-y* expression (Figure 1(i)) and IFN-y protein secretion (Figure 1(j)) than healthy controls, indicating that T2D-phages promote Th1 responses through DC activation.

### VLP and bulk metagenomics shows differential viral populations in T2D individuals

**Figure 2.**
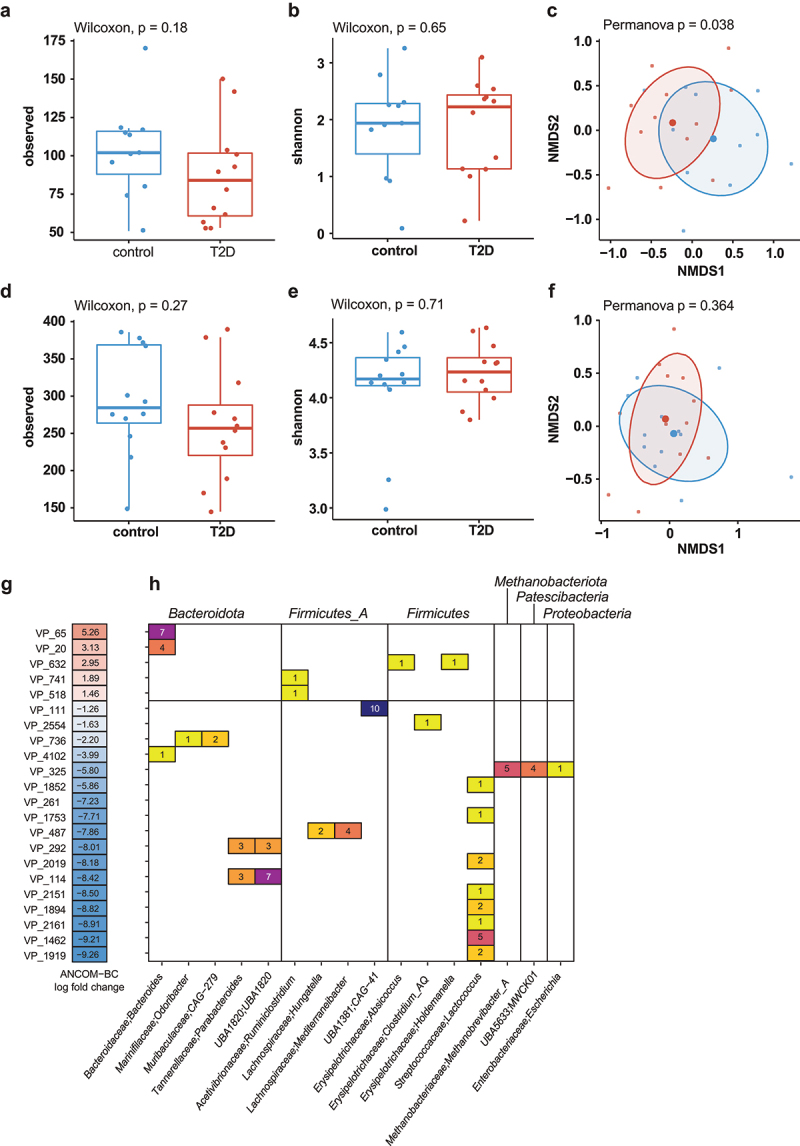
Differential viral populations in healthy vs T2D individuals.

To study phage populations in healthy and T2D groups, we isolated VLP DNA as well as total DNA (bulk) from the fecal samples of healthy and T2D individuals.^18,19^ Purified DNA was shotgun sequenced as previously described.^18,19^ After combining all viral sequences from the VLP and bulk fractions, we clustered them at 95% average nucleotide identity into viral populations (VPs) and took the largest from each VP to make viral clusters (VC) with vContact2.^41^ Richness or alpha-diversity (Shannon index) did not differ between controls and T2D in the VLP fraction (Wilcoxon ranked sum test, *p* > 0.05, Figure 2(a,b)). Beta diversity (Bray-Curtis), however, significantly differed between these groups (Permanova, *p* = .038, Figure 2(c)). Neither richness and alpha-diversity (Wilcoxon ranked sum test, *p* > .05, Figure 2(d,e)) or beta diversity (Permanova, *p* > .05, Figure 2(f)) differed in the bulk fraction of controls and T2D.

(a) Richness and (b) alpha-diversity (Shannon index) of viral clusters in healthy and T2D in the VLP fraction

(c) Non-metric multidimensional scaling (NMDS) on beta diversity (Bray-Curtis) of viral clusters in healthy and T2D in the VLP fraction

(d) Richness and (e) alpha-diversity (Shannon index) of viral clusters in healthy and T2D in the bulk fraction

(f) NMDS on beta diversity (Bray-Curtis) of viral clusters in healthy and T2D in the bulk fraction

(g) Differentially abundant (ANCOM-BC) viral populations in healthy and T2D.

(h) Host predictions for differentially abundant viral populations (g) in healthy and T2D. Colors and numbers indicate the number of viral contigs with a given host prediction within that VP.

We next determined which VPs were differentially abundant in either the controls or T2D groups with ANCOM-BC.^38^ In total, the 22 VPs were significantly differentially abundant (Benjamini-Hochberg-adjusted *p* ≤ .05), 17 of which in the controls (Figure 2(g,h)). Among these were a notably large number (*n* = 8) of predicted *Lactococcus* phages. Meanwhile, the two most differentially abundant viral populations in T2D were predicted to infect *Bacteroidota*.

In addition to bacteriophages, we also determined the abundance of other viruses. However, community profiling with Kraken2 with a National Center for Biotechnological Information (NCBI) reference sequence (RefSeq)-derived database showed that non-bacteriophage viral reads formed an extreme minority that was generally less than 0.001% of relative abundance (Figure S3). We therefore concluded that this provided too little information to accurately estimate non-bacteriophage viral differential abundance.

Among bacteria, we did not observe a difference in richness and alpha-diversity (Chao1 and Shannon, Wilcoxon ranked sum test, *p* > .05, Figure S4 a, b) or beta-diversity (Bray-Curtis, Permanova, *p* > .05, Figure S4c) between controls and T2D. Although this is in contrast to previous findings, this can likely be explained by the relatively small sample size. Between controls and T2D, 11 bacterial species were found to be significantly differently abundant (Benjamini-Hochberg-adjusted *p* ≤ .05, Figure S4d), of which 10 were more abundant in T2D. Notably, seven of the more abundant bacteria in T2D were from the *Bacteroides* genus, which aligns with the most abundant VPs in T2D that were predicted to infect bacteria of this genus.

## Discussion

The gut microbiome has been implicated in human disease, including T2D, with the majority of scientific endeavors focusing on bacterial members of the gut ecosystem. T2D is characterized by chronic low-grade inflammation with gut microbes and their metabolites are hypothesized to be underlying causal factors hereof.^7^ Less is known about inflammation-causing mechanisms of other members of the gut ecosystem in T2D. Here, we conceptually explored the immunogenic potential of human gut-derived phages and questioned whether phages from individuals with T2D had increased immunogenic potential compared to phages derived from healthy individuals. We show that phage solutions from T2D individuals have increased capacity to activate healthy donor dendritic cells and elicit an IFN-y response when co-cultured with same donor T-cells. In line with literature,^18,21^ we show that the gut virome of individuals with T2D differed from the viromes of healthy individuals. Specifically, viral populations predicted to infect *Lactococcus* were enriched in healthy donors whereas viral populations in T2D were predicted to infect *Bacteroidota*.

Recognition of phages by the mammalian immune system, with both pro- and anti-inflammatory properties, has been postulated by others before^42–46^ but was not studied in great detail until Gogokhia and coworkers reported increased IFN-y response by phages from individuals with inflammatory bowel disease as compared to healthy controls.^17^ Although our work is more exploratory in nature, our findings on the tendency to increase viral-like particles and increased immunogenic potential of phages from diseased as compared to healthy individuals, are in line with their findings. Nevertheless, our work needs dedicated follow-up studies using larger donor populations, this considering the significant variation in gut microbiota and virome compositions and the absence of single phages that correlate with T2D. We hence have not been able to consistently characterize specific phages with strong correlation to IFN-y in our small sample size. Additionally, although we aimed to reduce immunogenic factors in the phage solutions to the best of our capacity, we cannot exclude that remaining immunogenic factors exist.

Phages are nearly equally abundant as bacteria in the gut ecosystem and thus are a large component of fecal microbiota transplants; phages might hence contribute to immune activation in recipients of a healthy donor fecal microbiota transplantation (FMT), with subsequent consequences for (immune compromised) recipients and the success of this intervention. This has, to the best of our knowledge, never been addressed in human FMT trials but warrants consideration. Likewise, stressors on gut bacteria, including diet,^47^ can induce prophages that reside within the bacterial genomes, with subsequent release of virions with the potential to stimulate the human immune response. With a sharp focus on phages as alternatives for antibiotics,^48^ immune activation by phage therapeutics needs sharp attention to facilitate development of a safe and effective product.

Despite the limitations of our work, our results stress the physiological and therapeutic importance of studying phages as critical regulators of human health and disease; either by their regulatory actions on bacterial abundance or bacterial function or, as reinstated in our conceptual work, by their direct actions on the human immune system.

## Supplementary Material

Supplemental Material
