# Supplementary material for: Bacteriophages from treatment-naïve type 2 diabetes individuals drive an inflammatory response in human co-cultures of dendritic cells and T cells
Source: Gut Microbes. 2024 Jul 27;16(1):2380747. doi: 10.1080/19490976.2024.2380747 (PMC11285347; doi:10.1080/19490976.2024.2380747)
Supplement: Supplemental Material [file KGMI_A_2380747_SM2796.zip › Supplementary figures.docx]

**A Control B T2D**


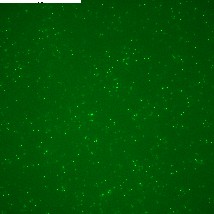


**50µm**


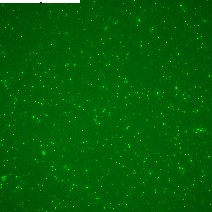


**50µm**

C

0.6

## TLR3

D

0.4

## TLR4

0.4

OD620

0.2

0.0

control T2D

neg control P(IC) 1 ug/mL

0.3027

**0.3**

0.2

OD620

0.1

0.0

control T2D

neg control LPS 100 ng/mL

0.1683

E

1.0

0.4163

0.8

0.6

OD620

0.4

0.2

0.0

## TLR5

control T2D

neg control Flagellin 100 ng/mL

F

1.5

1.0

OD620

0.5

0.0

## TLR9

control T2D

0.3760

neg control ODN2006 100 ug/mL

Figure S1

# A B

60 80

*

*

50

***IL-6* (norm. to *18S*)**

40 60

IL-6 (pg/ml)

30

4 40

3

2 20

1

0 0

DC monoculture

DC monoculture

neg control 1x106 VLPs

1x107 VLPs

**

**

*

1x108 VLPs

# C

6

4

IL-6 (pg/ml)

neg control 1x107 VLPS

LPS 0.1 ng/mL

** *

Figure S2

2

0

DC monoculture

Figure S3

100.0000%


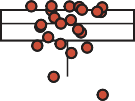

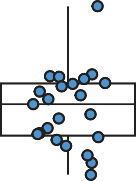


**VLP**


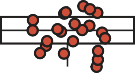

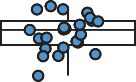


**Bulk**

relative abundance

10.0000%

1.0000%

0.1000%

0.0100%

0.0010%

0.0001%

bacteriophages other viruses bacteriophages other viruses

**A** 0.57 **B**

0.65

4200 3.9

4100

chao1

4000

3900

control T2D

**3.6**

3.3

shannon

3.0

2.7

control T2D

# C

0.50

Permanova p = 0.156

**D**

***Bifidobacterium catenulatum***

ANCOM−BC


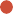

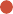


0.25

0.00

NMDS2

−0.25

−0.50


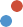


−0.5 0.0 0.5

NMDS1

***Bifidobacterium pseudocatenulatum***

***Bacteroides uniformis Bacteroides humanifaecis Bacteroides sp. HF−162 Bacteroides sp. A1C1 Bacteroides zhangwenhongii Bacteroides sp. CACC 737 Bacteroides faecium Actinomyces oris***

***Alistipes sp. dk3624***

−8 −4 0 4 8

log fold change

Figure S4
